# Pediatric COVID-19 in Argentina: a comprehensive analysis of disease and economic burden through official data and a systematic literature review

**DOI:** 10.3389/fped.2024.1352260

**Published:** 2024-03-28

**Authors:** Ariel Bardach, Silvina Ruvinsky, Carolina Moreno, Lucas Perelli, Moe H. Kyaw, Julia Spinardi, Carlos Fernando Mendoza, Carolina M. Carballo, Macarena Roel, Natalia Espinola

**Affiliations:** ^1^ Department of Health Technology Assessment and Health Economics, Institute for Clinical Effectiveness and Health Policy (IECS), Ciudad Autónoma de Buenos Aires, Argentina; ^2^ Centro de Investigaciones Epidemiológicas y Salud Pública (CIESP-IECS). CONICET, Ciudad Autónoma de Buenos Aires, Argentina; ^3^ Research Department, Hospital de Pediatría “Prof. Dr. Juan P. Garrahan”, Ciudad Autónoma de Buenos Aires, Argentina; ^4^ Vaccine Scientific Affairs, Pfizer Inc., New York, NY, United States; ^5^ Vaccine Medical Affairs, Emerging Markets, Pfizer Inc., Itapevi, Brazil; ^6^ Vaccine HTA, Value and Evidence, Pfizer Inc., Mexico City, Mexico; ^7^ Vaccine Medical Manager, Pfizer Inc., Buenos Aires City, Argentina

**Keywords:** pediatric COVID-19, disease burden, economic burden, Argentina, multisystem inflammatory syndrome, systematic review, meta-analysis

## Abstract

**Background:**

Limited data are available on the clinical impact and economic burden of COVID-19 in the pediatric population in Argentina. We aimed to estimate the disease and economic burden of COVID-19 on children and adolescents.

**Methods:**

We analyzed official national databases and conducted a supplemental systematic review of the published literature with meta-analysis in children aged 0–18. The period of interest was from March 2020 to August 2021, before the introduction of vaccination in this age group as a national strategic plan. In addition, we used a cost of illness analysis to estimate the direct medical costs associated with COVID-19. All costs are reported in US dollars 2023.

**Results:**

A total of 450,503 confirmed COVID-19 cases and 180 multisystem inflammatory syndrome (MIS-C) were reported in Argentina in the study period. Fourteen observational clinical studies were identified. The meta-analyses of severity level from hospital patients showed that according to different studies 15%–28% of cases were asymptomatic, 68%–88% were mild or moderate, and 3%–10% were severe or critical. About 28% of children had an underlying disease. In addition, the estimated economic burden associated with COVID-19 was 80 million dollars and 4 million dollars corresponded to MISC.

**Conclusion:**

Significant impact of COVID-19 on the healthcare system and substantial economic implications for the pediatric population in Argentina were identified. The findings should help policymakers to make informed decisions and allocate resources effectively.

## 1 Introduction

Since the early 2020s, COVID-19 has spread rapidly worldwide, causing a global pandemic (1). The severe acute respiratory syndrome associated with SARS-COV2 resulted in numerous deaths and had a significant economic impact worldwide (2). The global COVID-19 pandemic has directly or indirectly impacted the pediatric population. Pediatric patients with SARS-COV2 are often asymptomatic or have mild symptoms, with a lower risk of hospitalization and life-threatening complications (3). However, during the pre-Omicron variants circulation, a similar age distribution was observed in hospitalization rates, affecting groups of children under five and adolescents aged 12–14 (4). In Argentina, the pediatric population represented roughly 11% of all confirmed patients. Case fatality in this age group was estimated to be 0.054% (5). On August 21st, the pediatric population began vaccination (6).

Detailed insights on outpatient care, hospitalization, disease severity, potential complications, resource utilization patterns, and the corresponding financial implications remain scarce within the country. The available official databases solely provide information on numerical case tallies, admissions to the Pediatric Intensive Care Unit (PICU) admissions, mechanical ventilation needs, and fatality statistics. Some case series have described pediatric patients' clinical and epidemiological characteristics (7, 8). One study examined the impact of different variants on children under 18 years of age (8). Managing pediatric COVID-19 cases involves a multidisciplinary approach, including pediatricians, infectious disease specialists, and critical care specialists. The management of severe COVID-19 cases in children is similar to that in adults, with oxygen therapy, mechanical ventilation, and extracorporeal membrane oxygenation (ECMO) in severe cases. In addition, managing MIS-C involves using immunomodulatory therapy, including intravenous immunoglobulin (IVIG), corticosteroids, and biological agents (9, 10).

Estimating the disease and economic impact of COVID-19 on the pediatric population allows policymakers to make informed decisions and allocate resources effectively (11). This is especially crucial in healthcare systems with significant budget constraints and challenges related to Universal Health Coverage (12). Regarding economic studies of COVID-19, impact and cost-effectiveness studies of COVID-19 vaccination for the region have been published, such as those by Augustovsky et al. (13) and Taborda et al. (14) However, specific studies on the economic burden of the disease in the pediatric population have yet to be published.

We aimed to evaluate the disease and economic burden of COVID-19 in the pediatric population from a health system perspective in Argentina from March 2020 to August 2021 before nationwide pediatric COVID-19 vaccination.

## 2 Material and methods

We used the national datasets to analyze the health burden of pediatric COVID-19 disease. We searched national and subnational databases that reported the impact of COVID-19 on the pediatric population. The primary and most comprehensive source of information was the official information from National Surveillance System reports from the Argentine Ministry of Health, which, however, only provided data on the number of cases, PICU admissions, mechanical ventilation requirements, and fatalities (7). Second, to provide complementary information on parameters that were not found in national databases, such as severity of disease, MIS-C cases, hospital and PICU stay, and use of resources, we conducted a systematic literature search from March 1, 2020, to August 9th, 2021, in Argentine children and adolescents from 0 to 18 years old. Data on epidemiological surveillance from Argentina during the same period were analyzed using the Cochrane methods and the 2020 Preferred Reporting Items for Systematic Reviews and Meta-Analyses (PRISMA) (15) statement for reporting results (15). Two reviewers independently performed title-abstract screening on all selected studies, followed by a comprehensive assessment of the complete text of the designated articles. Disagreements were resolved by consensus of the entire research team. Interrater reliability was evaluated with the Kappa coefficient of agreement. The risk of bias in the studies was assessed using the checklist developed by the United States National Heart, Lung, and Blood Institute, which classifies studies as having high (Poor), moderate (Fair), and low (Good) risk of bias (16). We applied an arc-sine transformation to stabilize the variance of proportions following the Freeman-Tukey variant of the arc-sine square root of transformed proportions method (17). As for eligibility criteria, comparative and non-comparative study designs were included regardless of publication status, publication year, or language. Studies without a clear denominator, narrative reviews, and articles with unavailable full-text and systematic reviews (SRs) were excluded; however, the reference lists of SRs on the subject were examined for relevant studies. We searched the Latin-American and Caribbean Health Sciences Literature (LILACS), Medline, Embase, SciELO, Cochrane Library database, and WHO Database publications on MIS-C and SARS-CoV-2 and CRD York Prospero and preprint databases (ArXiv, BiorXiv, medRxiv, search.bio Preprint). We also searched the proceedings of international, national, or regional (LAC) scientific meetings from March 1st, 2020, to August 9th, 2021. The search strategies utilized can be found in Supplementary Material.

We explored epidemiological outcomes regarding disease burden (total cases according to WHO COVID-19 classification), demographic data, underlying diseases, hospitalization rate, PICU admission, complication (MIS-C, Kawasaki Disease), case fatality ratio, and health resource usage (oxygen requirement, mechanical ventilation, and treatment). Studies regarding long COVID-19 or RCTs were excluded.

### 2.1 Study selection, data extraction, and assessment of the risk of bias in included studies

We used COVIDENCE Software (18) for the initial phases of the systematic review (18). We also explored the reports of passive surveillance report systems from the Pan American Health Organization (PAHO) and LAC countries and the Argentine Ministry of Health. Potentially eligible studies were retrieved in full text, and two reviewers independently extracted and assessed the risk of bias. Disagreements were also resolved by discussion among the review team members. For data extraction, an online spreadsheet was used. The research team extracted study characteristics (type of publication, year published, authors, geographic location, study design including the risk of bias method), population characteristics, and outcomes (incidence rate, specific mortality, and fatality rate). Authors of articles were contacted when necessary for supplementary information. The risk of bias in observational studies and the control arm of trials was assessed using a checklist developed by the United States National Heart, Lung, and Blood Institute (19), which classifies studies as high (Poor), moderate (Fair), and low (Good) risk of bias. For the assessment of cross-sectional studies, the tool comprises 14 items, while the tool for case-series studies entails nine domains (19). The evaluation was carried out independently by peer reviewers from the research team. We resolved discrepancies by consensus of the entire research team. The risk of bias is shown in Supplementary Tables S1 and S2.

### 2.2 Data synthesis

We conducted proportion meta-analyses. We applied an arc-sine transformation to stabilize the variance of proportions (Freeman-Tukey variant of the arc-sine square-root of transformed proportions method), *y* = arcsine[√(*r*/(*n* + 1))] + arcsine[√(*r*/(*n* + 1)/(*n* + 1)], with a variance of 1/(*n* + 1), where *n* is the population size (17). Pooled proportions were calculated as the back-transformation of the weighted mean of the transformed proportions, using inverse arcsine variance weights for the fixed and random-effects model. We applied DerSimonian-Laird weights for the random-effects model where heterogeneity between studies was found. We calculated the I^2^ statistic to measure the proportion of the overall variation attributable to between-study heterogeneity. The R software package *meta* and its functions *metamean*, *metaprop*, and *forest.meta*, and STATA 14.0 were used. Extracted data were synthesized using descriptive and meta-analytic approaches using the outcome measures (RRs or Mantel-Haenszel ORs) or (Peto ORs) for dichotomous data. We reported mean difference (MD) and 95% confidence interval (CI) for all outcomes for continuous data. We used simple descriptive statistics whenever not estimable to calculate association measurements.

### 2.3 Economic burden of COVID-19

The economic burden per patient associated with COVID-19 was calculated using the cost of illness analysis through the micro-costing approach (20). This method consisted of identifying health resources, rates of use, quantities and unit costs. These components were then multiplied to obtain the expected cost per health resource. We used a healthcare system perspective. Only direct medical costs were considered in this analysis, including drug acquisition and administration costs, disease monitoring (laboratory tests and image), hospitalization (General Ward, PICU, and PICU with mechanical ventilation), and medical consultations. In Argentina, the healthcare system is decentralized and fragmented into three subsectors: public, social security, and private. The social security sector is the largest and provides healthcare coverage to approximately 46% of the Argentine population; the private sector covers 18%, and approximately 36% is covered by the public sector (21).

The identification of healthcare resources, their usage rates and quantities used for disease management were estimated using the WHO clinical practice guideline for COVID-19 management and estimated by severity (mild, moderate, severe, and critical) (22). In addition, a process of validation of the data obtained through consultation with local experts was carried out. A detail on the expected healthcare resource utilization by level of severity can be found in Supplementary Table S4. On the other hand, the unit costs of health resources were estimated for each subsector (social security, public sector and the private sector), and obtained from the Unit Cost Basis of the Institute for Clinical Effectiveness and Health Policy (23). A detail on the unit costs of health resources by subsector can be found in Supplementary Table S5. Regarding the drugs included in the estimation, only non-steroidal anti-inflammatory Drugs (NSAIDs) were included in mild and moderate cases, while in severe cases, corticosteroids and antibiotics were included. In addition to the medications described in severe cases, anticoagulants were also included in critical cases. The costs of the drugs were obtained from the *AlfaBeta Group* pharmaceutical manual (24), and the doses of each drug were calculated according to what is recommended for an adolescent weighing 55 kg (For more detail, see Supplementary Table S3).

The expected total cost by severity level and healthcare subsector resulted from the sum of the products of the three components (rates of use, quantities, and unit costs). To obtain the total cost associated with COVID-19, we used a weighted average by the rate of coverage of each subsector and the distribution of cases of COVID-19 by severity. In the ministerial databases, there was an absence of data pertaining to the distribution of COVID-19 case severity. Furthermore, the outcomes derived from the meta-analyses are not applicable due to an inadequate level of granularity and the fact that central estimates do not cumulatively amount to 100%. See section 4.3. Hence, we decided to use the Gentile study (8), which analysed data from a large sample of patients in paediatric centres in the country, and presented good quality. This study presented that of the total COVID-19 cases, 25.0% were asymptomatic cases, 70.6% were mild cases, 1.7% moderate cases, 1.7% severe cases, and 1% critical cases. The assessment of the economic burden of COVID-19 focused on mild, moderate, severe and critical patients, assuming that the asymptomatic patients did not incur costs for the health system. Therefore, the redistribution by severity of symptomatic cases is 94.1% mild cases, 2.3% moderate cases, 2.3% severe cases and 1.3% critical cases.

In addition, we estimated the cost of Pediatric Inflammatory Multisystem Syndrome (MIS-C), a complication that occurred significantly in the pediatric population with COVID-19. The micro-costing of this complication was carried out using national (25) and international (9) MIS-C management guidelines and the opinion of experts in the area belonging to the work team. In the Supplementary Table S6 presents the information related to the expected healthcare resource utilization, and the unit cost per healthcare sector associated with estimating COVID-19 cost and MIS-C. The percentage of COVID-19 cases that could develop MIS-C was retrieved from the Argentine Ministry of Health (25), estimated at 0.04%. All costs were estimated as of May 2023 and reported in USD with an exchange rate of 232.21 ARS per USD (26).

### 2.4 Other vaccine-preventable diseases

Complementary, we explored the disease and economic burden of other preventable diseases (Influenza, Invasive Pneumococcal, Meningococcal disease, Chickenpox, and Rotavirus) before vaccine introduction in NIP (National Immunization Program) for reference only. It is important to highlight that disease comparisons are inadequate due to differences in burden, epidemiological settings, age prevalence, surveillance system, and agent characteristics, among others. We retrieved data from the Argentine Ministry of Health and the National Surveillance System, and an exhaustive literature review was conducted to compile data on health outcomes. We also had the input of a local expert on pediatric infectious diseases. A complementary literature search was conducted using the PubMed and Lilacs databases to estimate the total costs associated with the selected diseases (See Supplementary Material). Studies of economic burden or direct medical costs were identified for each vaccine-preventable disease in Argentina. The costs were updated for inflation using the Consumer's Price Index obtained from the Institute of Statistics and Census (INDEC, acronym in Spanish) to April 2023 (27). Costs are reported in US dollars for 2023 (26).

## 3 Results

We present data from the official surveillance system and complement it with the information from the systematic literature review.

### 3.1 Data analysis from official information

Table 1 shows the information analyzed from national statistics databases between March 2020 and August 2021. In general, 450.503 confirmed pediatric cases were reported, and the median age was 15 (95% CI: 10–18 years). Specifically, 610 patients with COVID-19 required intensive care (1.35 per 1,000 patients), and 219 required mechanical respiratory support (0.48 per 1,000 patients).

**Table 1:** Health ministry data on 450.503 patients in the march 2020—August 2021 period.

Age (year)	0–4			5–9			10–14			15–19			Total
** **	F	M	NR	F	M	NR	F	M	NR	F	M	NR	
Cases	20,401	22,598	2,601	31,111	32,743	795	52,393	50,979	1,729	124,741	108,405	2,007	450,503
PICU	79 (0.39%)	126 (0.56%)	102 (3.92%)	45 (0.14%)	37 (0.11%)	2 (0.25%)	37 (0.07%)	47 (0.09%)	0	70 (0.06%)	62 (0.06%)	3 (0.15%)	610
MV	28 (0.14%)	47 (0.21%)	25 (0.96%)	21 (0.07%)	11 (0.03%)	0	16 (0.03%)	17 (0.03%)	0	25 (0.02%)	27 (0.02%)	2 (0.1%)	219
Deaths	35 (0.17%)	36 (0.16%)	27 (1.04%)	20 (0.06%)	15 (0.05%)	0	11 (0.02%)	23 (0.05%)	0	30 (0.02%)	39 (0.04%)	2 (0.1%)	238
Mortality rate (per 100,000)	2.09	2.04		1.12	0.79		0.64	1.26		1.77	2.19		

In parentheses, we present the percentage of confirmed cases in each age group and sex who experienced the event. The mortality rate was estimated considering the total country population for that group.

PICU, Pediatric Intensive Care Unit; MV, mechanical ventilation; F, female; M, male; NR, not reported.

The case fatality ratio was 0.05%, with 238 deaths observed. A bimodal pattern can be observed in the age distribution of deaths, with higher incidence rates between 0 and 4 years of age (2.85 per 100,000 inhabitants) and between 15 and 19 years of age (2.04 per 100,000 inhabitants) (Table 1).

### 3.2 Data analysis from the systematic review

**Figure 1:**
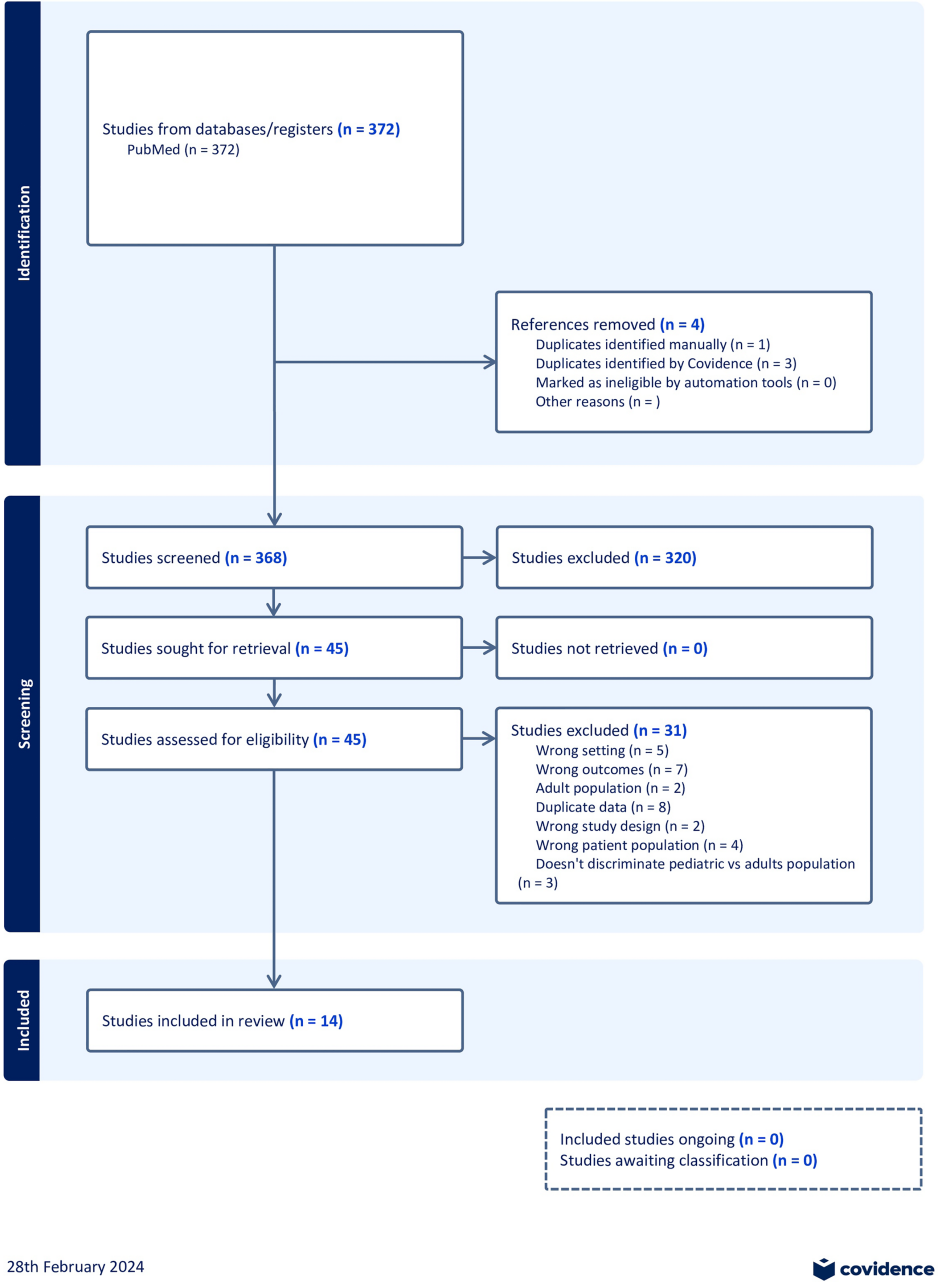
PRISMA 2020 study diagram.

We searched multiple databases comprehensively to identify relevant studies for inclusion in this systematic review. The search was restricted to studies published with patient data between March 2020 and August 2021. After removing duplicates, we screened 368 studies based on their titles and abstracts and 45 full-text articles for eligibility. In total, 14 studies met the eligibility criteria. A flow diagram outlining the study selection process, as recommended by the Preferred Reporting Items for Systematic Reviews and Meta-Analyses (PRISMA) guidelines, is provided in Figure 1. A summary of the studies included in this meta-analysis is presented in Table 2.

**Table 2:** Description of the argentinean pediatric COVID-19 studies included.

Author	Published year	Setting	Main outcomes assessed	Type of study	Type of population (median age in months; IQR)
Aprea, V	2021	Out-of-hospital care setting in a Febrile Emergency Unit of suspected COVID-19 patients	Cases, diagnosis	Case series	Pediatric outpatients (70; NA)
Brizuela, M. E	2022	Correlation of SARS-CoV-2 viral load and clinical evolution of pediatric patients in a General Hospital from Buenos Aires, Argentina	Cases, diagnosis, severity, LOS, resources utilization	Cohort	Pediatric inpatients (60; 14–108)
Capra, D	2021	Experience at the Department of Pediatrics of a private facility in the Metropolitan Area of Buenos Aires during the COVID-19 pandemic	Cases, diagnosis, severity	Case series	Pediatric outpatient and inpatients (82; 20–147)
Ferraro, D	2021	Epidemiological characteristics according to SARS-CoV-2 pandemic progression in a high complexity pediatric hospital in Argentina: a descriptive study.	Cases, diagnosis, severity, LOS, resource utilization	Case eeries	Pediatric outpatient and inpatients (83; 33–144)
Gentile, A	2022	COVID-19 in children: correlation between epidemiologic, clinical characteristics, and RT-qPCR Cycle threshold values	Cases, diagnosis, severity, complications, resource utilization	Cohort	Pediatric inpatients (85; 23–165)
Gentile, A	2022	A multicenter study of confirmed COVID-19 cases: preliminary data on 2,690 pediatric patients in Argentina	Cases, diagnosis, severity, complications, resource utilization	Cohort	Pediatric outpatient and inpatients (67; 15–135)
Golemba, M. D	2022	Comparison of SARS-CoV-2 viral load in asymptomatic and symptomatic children attended in a referral public pediatric hospital in Argentina	Cases, diagnosis, admission	Cross-sectional	Pediatric outpatient and inpatients (60, 16–129)
Gómez, S	2021	Clinical and outcome characteristics of children with SARS CoV-2 infection in a tertiary-care hospital: a cohort study	Cases, diagnosis, severity, resource utilization, complications	Cohort	Pediatric outpatient and inpatients (75; 22–143)
Medina, M. L	2021	Prevalence and epidemiological characterization of SARS-COV-2 in children of the Chaco, Argentina	Cases, diagnosis, admission	Case series	Pediatric outpatient and inpatients (84; NA)
Raiden, S	2021	Children hospitalized for COVID-19 during the first winter of the pandemic in Buenos Aires, Argentina	Cases, diagnosis, Admission	Case series	Pediatric inpatients (48; 8–134)
Rosanova, M. T	2021	Pediatric Inflammatory Multisystem Syndrome Associated With SARS-CoV-2: a retrospective cohort study from Argentina	Cases, diagnosis, complications	Cohort	Pediatric inpatients (104; 61–126)
Rubinos, M	2022	Experience in pediatric patients with COVID-19 during the first pandemic wave	Cases, diagnosis, severity, complications, resource utilization	Case Series	Pediatric outpatient and inpatients (66; 13–132)
Vainstein, E	2022	Multicentre observational study on multisystem inflammatory syndrome related to COVID-19 in Argentina	Cases, diagnosis, complications	Case Series	Pediatric inpatients (72; 37–117)
Gentile, A	2023	Comparison of epidemiologic and clinical COVID-19 profiles in children in Argentina, during circulation of original and variant (Alpha, Gamma and Lambda) Strains	Cases, diagnosis, severity, complications, resource utilization	Cohort	Pediatric inpatients (90; 25–152)

IQR, interquartile range.

### 3.3 Risk of bias

Seven of the included studies were assessed using the NIH tool for evaluating observational studies for case series (Supplementary Table S1). Among these studies, only one was rated as “good” regarding methodological quality, and the remaining were rated as “fair”. Five other studies were assessed using the NIH tool for Cohort and Cross-Sectional Studies (Supplementary Table S2). Each of these studies was assessed across the 14 domains evaluated by the tool. Only one of these studies was rated as “good” regarding methodological quality, three as “fair,” and one as “poor.”

### 3.4 Clinical characteristics of the study population

**Figure 2:**
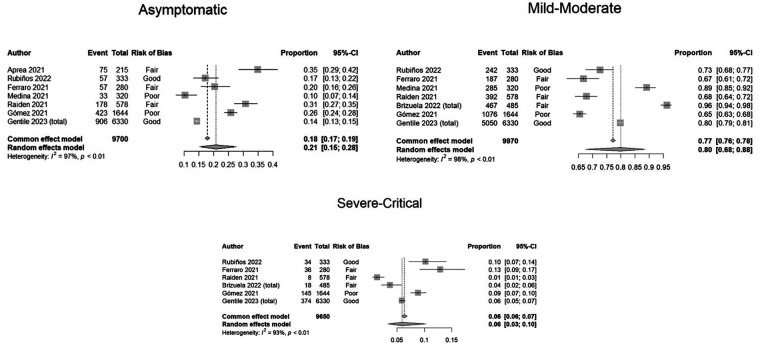
Forest plots showing proportion meta-analyses of clinical severity according to WHO classification in COVID-19 pediatric patients assessed in hospitals. Argentina, 2021.

A total of 14 studies with 10,620 patients with SARS-CoV2 infection were included. Three included 299 patients with MIS-C associated with SARS-CoV2 infection following the Centers for Disease Control and Prevention (CDC) or World Health Organization (WHO) diagnosis criteria. Demographic data, clinical manifestation, outcomes, treatment, and use of resources were analyzed and reported in Table 3. The mean age from the meta-analysis of the included studies was 5.7 years (95% CI: 4.7–6.8), compared to the median age analyzed in the official national data of 15 years (95% CI: 10–18 years). After analyzing all the relevant clinical literature available, we conducted several meta-analyses on the severity of COVID-19 in pediatric patients. It was ensured that the study populations were comparable before conducting the analyses. According to the WHO classification, Figure 2 illustrates the proportion of clinical severity in COVID-19 pediatric patients evaluated in hospitals during the pre-vaccination era.

**Table 3:** Meta-analysis (*N* = 14 studies) of demographics, clinical characteristics, and use of resources.

Variables	Studies (*) *n*/*N*	Cases/total *n*/*N*	Pooled proportion (%) (95% CI) from meta-analyses
Age, month, median (IQR)	10/14	10,085/10,620	68.73 (56.6–83.5)
Male sex	11/14	5,315/10,405	51 (50–52)
Underlying diseases	10/14	2,742/10,323	28 (19–38)
MIS-C incidence	5/14	171/9,067	2 (1–4)
LOS, median (IQR)	7/14	3,521/10,629	6 (3.7–9.4)
PICU admission	6/14	270/8,788	8 (3–20)
Mortality	6/14	55/8,997	1 (0–1)

IQR, interquartile range; PICU, Pediatric Intensive Care Unit; LOS, length of stay.

We found that 21% (95% CI: 15–28) were asymptomatic, 80% (CI: 68–88) mild-moderate, and 6% (95% CI: 3–10) severe-critical. (See Figure 2, meta-analyses). Important levels of heterogeneity were found.

### 3.5 Hospitalization, PICU admission, complications, and need for mechanical ventilation

Concerning the need for hospitalization, although the meta-analysis of proportions showed a need for hospitalization in 24% of patients (95% CI: 11–44), there was a marked disparity between the articles included. In the meta-analysis of proportions of included studies, PICU admission was 8% (95% CI: 3%–20%), while the proportion of patients requiring mechanical ventilation was 1% (95% CI: 0%–5%). This information is presented in Table 3. In addition, In the Supplementary Material, we presented information on the forest plots of frequencies of these variables analyzed.

Analysis of the pooled data from 11 studies involving 10,444 patients revealed respiratory symptoms in 31% of cases (95% CI: 21–42) (See Supplementary Table S8). Fever, one of the hallmark symptoms of COVID-19, was observed in 60% of cases (95% CI: 45–73). Gastrointestinal symptoms were reported in 15% of COVID-19 patients [95% confidence interval (CI): 8–25]. Similarly, neurological symptoms were reported in 10% of patients (95% CI: 6–18) based on data from eight studies involving 9,366 individuals. Skin rash was observed in 5% of cases (95% CI: 1–16) according to data from six studies, which included 2,999 individuals. Furthermore, comorbidity related to an increased risk of severe disease was reported in 28% of the cases. Previous respiratory disease was observed in 6% (95% CI: 4–8), onco-hematological conditions in 5% (95% CI: 2–10), neurological comorbidities in 4% (95% CI: 3–7), and genetic comorbidities were reported in 2% (95% CI: 1–4), respectively, based on data from four studies, with a total of 1,169 pediatric patients. Lastly, other comorbidities were found in approximately 6% of patients (95% CI: 3–11) based on data from seven studies involving 7,791 individuals.

### 3.6 MIS-C related to COVID-19: clinical characteristics and use of resources

MIS-C was reported in a total of 372 patients from all studies included. Meta-analysis was performed on 299 pediatric patients with MIS-C whose characteristics were described. The median age was 83.5 months (95% CI: 67–104 months), male sex was 52% (95% CI: 35%–68%), and underlying diseases were observed in 16% (95% CI: 12%–21%). Pooled length of stay was nine days (7.19–11.13 days). 37% (95% CI: 32%–43%) were admitted to PICU. Most patients received gamma globulin alone 90% (95% CI: 60%–98%) or with corticosteroids 61% (95% CI: 55%–66%). Only one study reported the use of tocilizumab in 6/176 patients (3%) and 39/176 (22%) of the patients received anticoagulants. The case fatality ratio was 5/299 (1.6%).

### 3.7 Healthcare costs of COVID-19

Table 4 presents the number of COVID-19 cases, the total healthcare system cost per case by the severity of COVID-19, and the number of MIS-C cases and the cost per case. In addition, the results of the overall economic burden of COVID-19 and MIS-C are estimated and presented.

**Table 4:** Healthcare costs of COVID-19 per case in the march 2020—August 2021 period (USD 2023).

	Number of cases	%	Cost per case, $	Total cost
Mild COVID-19	424,073	94.1%	$67.59	$28,665,207.4
Moderate COVID-19	10,211	2.3%	$1,077.47	$11,002,499.9
Severe COVID-19	10,211	2.3%	$2,132.40	$21,774,804.2
Critical COVID-19	6,007	1.3%	$3,135.66	$18,834,972.5
All COVID-19^a^	450,503	100%	$178.20	$80,277,484.0
MIS-C	180	0.04%	$22,902.45	4,122,441.0
Total				$84,399,925.0

Mild: symptomatic COVID-19, Moderate: COVID-19 with pneumonia symptoms, Severe: COVID-19 with symptoms of severe pneumonia, Critical: COVID-19 with symptoms of ARDS.

^a^ Total cost weighted by severity level.

The total economic burden associated with COVID-19 in the pediatric population is around 80 million dollars, and the burden associated with MIS-C is approximately 4 million. As expected, the cost per COVID-19 case increased by the degree of severity; they ranged between 67.6 dollars (mild case) to 3,135.7 dollars (critical case)). The critical case included costs for treating Acute Respiratory Distress Syndrome (ARDS). Additionally, the cost of a MIS-C case is 22,749.8 dollars. In the Supplementary Table S9 shows the economic burden associated with COVID by subsector.

### 3.8 Other vaccine preventable diseases

Regarding other immune preventable diseases before vaccination, pneumococcal disease posed a significant disease burden among pediatric patients in Argentina, with pneumococcus being the primary cause of bacteremia and bacterial meningitis. Before PCV13 NIP inclusion in 2012, consolidating pneumonia incidence in children under five years old was estimated at 1,256 per 100,000 inhabitants, with a hospitalization rate of approximately 65% (28). The mortality rate for pneumonia of all causes in this age group was 1.1%. Pneumococcal bacteremia and sepsis incidence among children under two years old was 98.6 per 100,000, with a lethality rate of 1.5%. Pneumococcal meningitis incidence in children under five was 3.49 per 100,000, with a mortality rate of approximately 14%. Invasive meningococcal disease also had a notable impact, mainly affecting children under five, with an annual incidence ranging from 170 to 300 cases. Infants under 12 months experienced the highest incidence (13.2 cases per 100,000). Invasive meningococcal disease carried a case fatality rate of 10% (29). Influenza vaccination was incorporated into the National Immunization Schedule in 2011. 2,411 deaths were attributed to pneumonia and influenza between 2002 and 2009, see Supplementary Table S10, and hospitalizations reached an average of 7,868 to 22,994 per year during that period (30). Before the introduction of mandatory rotavirus vaccination, acute diarrhea affected 1,100,000 to 1,250,000 individuals annually in Argentina, with children under five accounting for 45%–50% of cases (31, 32). Chickenpox recorded an estimated 150,000 to 180,000 cases annually before introducing the vaccine to the immunization schedule, but underreporting suggested a higher incidence of approximately 350,000–400,000 cases annually (33, 34). The average cost per case of pneumococcal disease was between $1,026.13 and $2,028.16, and rotavirus was between $36.48 and $96.30. The cost per hospitalized influenza case was between $344.40 and $623.23. The cost per outpatient chickenpox case was between $47.26 and $71.55. Finally, the cost per inpatient chickenpox case was between $721.58 and $2,085.31. In the Supplementary Table S11, the cost results for this section are presented in more detail.

## 4 Discussion

This is the first pediatric study encompassing published literature and official data on pediatric COVID-19 disease and economic burden in Argentina before the vaccine introduction. This study highlights the significant impact of COVID-19 on the healthcare system and the substantial economic implications associated with COVID-19 in the pediatric population. It suggests the need for effective control measures to reduce the disease burden.

During the evolution of the pandemic periods and the circulation of different SARS-CoV-2 variants, hospitalization criteria were changed to be more restricted, recommending it only to patients younger than six months old, with comorbidities or severe disease (8). Therefore, it is difficult to analyze and establish the real length of stay reported from different studies, probably overestimated in the first period from pandemia. Differences observed in median age between Ministry of Health reports and included studies can be attributed to the fact that official information came from positive pediatric cases reported, while the pediatric studies are all centered on hospitalized children. Most children and adolescents presented mild symptoms. However, acute severe and critical cases and severe complications related to COVID-19 infection, MIS-C were reported with high impact in the pediatric population in our study, similarly reported in other countries (35). Pediatric studies show that patients with comorbidities have more risk of severe disease; we found underlying diseases in 28%, as reported by Niño and cols (36). Other pediatric series from Latin America and other countries showed similar data (37). Moreover, studies including the pre-vaccination period have shown similar data about severe clinical presentation, MIS-C, hospitalization, and resource use.

We found that between March 2020 and August 2021, when variants Alpha, Beta, Gamma, and Delta circulated, 450.503 confirmed pediatric cases were reported in Argentina, mainly in the age range of 13 to 17. Deaths were 2.9/100,000 children aged 0–4 and 2.0/100,000 in ages 15–19. Lethality ranged from 0.02 in ages 15–19 to 0.17% in 0–4 years, and 28% (19–38) had an underlying medical condition. The pooled median length of stay was 6 IQR (3.7–9.4). The pooled proportion of PICU admission was 8%, 95% CI: 3%–20%. MIS-C pooled percentage was 2% in hospitalized children (1%–4%). Global mortality among hospitalized of all ages was 1%.

The studies in our review found a similar mortality rate than reported in national databases, with a ratio of 0.05 and a central estimate from the meta-analysis of proportions of 0.01 (95% CI: 0.00–0.03).

In economic terms, this study reveals that, before vaccination implementation, total direct medical costs related to COVID-19 in the pediatric population represented approximately 0.1% of total health spending in the country. Although the costs per case show a substantial difference by severity. In turn, it can be observed that the costs per case of COVID-19 requiring hospitalization (moderate, severe and critical) were around four times higher compared to the cost per case hospitalized for influenza or chickenpock pre-vaccination found in the literature. These results make it possible to identify the use of resources and the cost generated by the treatment of the disease for the health system, making it possible to measure the problem, and provide information to decision makers for efficient spending and resource use policies.

This study has several strengths contributing to its validity and reliability. Firstly, the completeness of the search is a strength of this study. We thoroughly searched official sites to gather relevant information on the impact of COVID-19 on the pediatric population. The use of multiple sources of information ensures that the study is comprehensive and provides a more accurate representation of the impact of COVID-19 on the pediatric population. Secondly, conducting meta-analyses of proportions to synthesize the evidence is another strength of this study. The use of meta-analyses allows the authors to combine data from multiple studies to provide a more accurate estimate of the impact of COVID-19 on the pediatric population on important outcomes such as severity, length of stay, hospitalization rates, MIS-C incidence, and comorbidities. This approach increases the statistical power of the study and provides a more robust estimate of the impact of COVID-19 on the pediatric population. In addition, this is a pioneering study to estimate the direct medical cost of COVID-19 in the pediatric population in the Latin American region.

The study presents certain limitations to be considered, including the need for more analysis on disseminating variants with different degrees of severity within the region. Also, disease severity in identified papers varied in its definition and frequency. Furthermore, an analysis of the period that followed the vaccination process, during which less virulent strains began to circulate, is warranted. On the other hand, while an attempt was made to conduct a detailed analysis to estimate the direct medical cost of COVID-19, the fragmented and decentralized nature of Argentina's healthcare system may result in estimates not fully capturing all these heterogeneities. For future projects, considering a Delphi Panel with experts from different contexts, as well as the utilization of real-life data, could be beneficial.

Epidemiological studies often exhibit more heterogeneity in frequency and magnitude than experimental studies conducted under controlled conditions. As a result, it is prudent to conduct meta-analyses using the random-effects model, which accounts for variability across studies and yields broader, more conservative confidence intervals. Especially in cases of high heterogeneity, these confidence intervals are of most importance and could be chosen over point estimates (38).

Last, in this first wave, stringent and prolonged isolation measures in Argentina impacted viral co-circulation. Immunization has led to a reduction in both morbidity and mortality rates, as well as a decrease in the incidence of complications.

The burden of the other diseases that can be prevented by immunization has been significant in terms of health and economics in the pre-vaccination era. Including routine childhood vaccination in the Immunization National Program has led to improvements in public health and a significant reduction in economic burden, both directly and indirectly.

The present study provides a comprehensive analysis of the impact of COVID-19 on the pediatric population in Argentina, including outpatient care, hospitalization, use of resources, and related costs. When we analyze the impact of COVID-19 disease, the incidence rate is higher in adolescents compared to younger children. Similar results were reported in the USA (39). However, a bimodal pattern can be observed in the age distribution of deaths, with higher rates between 0 and 4 years of age (2.85 per 100 thousand inhabitants) and between 15 and 19 years of age (2.04 per 100 thousand inhabitants). These data indicate children are also at risk for severe COVID. This study is essential for informing public health policies and interventions aimed at mitigating the impact of COVID-19 on the pediatric population in Argentina.

Despite the growing body of literature on the impact of COVID-19 on the pediatric population, several unanswered questions still require further research. Firstly, there is a need for more longitudinal studies that follow children over time to understand better the long-term effects of COVID-19 on their health and well-being (long-COVID). Secondly, there is a need for more studies that focus on the impact of COVID-19 on vulnerable populations, such as children living in poverty or with underlying medical conditions. Thirdly, there is a need for more studies examining the impact of COVID-19 on children's mental health, including the impact of lockdowns and social distancing measures on their emotional state and lifestyle. Fourthly, there is a need for more studies that examine the impact of COVID-19 on the use of healthcare resources and related costs in the pediatric population. Finally, there is a need for more studies that examine the impact of COVID-19 vaccines on the pediatric population. Nevertheless, the findings from this study should allow policymakers to make informed decisions for preventive and treatment management and allocate resources effectively.

## Data Availability

The raw data supporting the conclusions of this article will be made available by the authors, without undue reservation.
