# Supplementary material for: Pediatric COVID-19 in Argentina: a comprehensive analysis of disease and economic burden through official data and a systematic literature review
Source: Front Pediatr. 2024 Mar 28;12:1352260. doi: 10.3389/fped.2024.1352260 (PMC11007211; doi:10.3389/fped.2024.1352260)
Supplement: Supplementary file 1 [file Datasheet1.pdf]

Supplementary material

Index

Search Strategies 2
Risk of bias 5
Table 1. Risk of bias for Case Series included in the systematic review 5
Table 2. Risk of Bias for Cohort and Cross-Sectional Studies 5
Details on estimation of direct medical cost of COVID and MISC 6
Table 3. Drugs and dosage 6
Table 4. Expected healthcare resource utilization (HCRU) for the treatment of COVID-19 case by level of severity 6
Table 5. Unit costs of healthcare resources used for the treatment of COVID-19 by healthcare sector in Argentina, expressed in USD 2023 7
Table 6. Healthcare resource utilization (HCRU) and unit costs (USD 2023) per healthcare sector used for the treatment of MIS-C 8
Detail of the research on the disease and economic burden of other immunopreventable diseases pre-vaccination era 9
Mortality 10
Forest plots showing proportion meta-analyses (N=14 studies) of demographics, clinical characteristics, and use of resources 14
Total economic burden of COVID-19 case by healthcare sector 17
Results of of other immunopreventable diseases pre-vaccination era 17
Table 10. Deaths and Discharges of other immunopreventable diseases pre-vaccination era 17
Table 11. Costs of other immunopreventable diseases pre-vaccination era, expressed in USD 2023 18

Search Strategies

PubMed

(Ncov*[tiab] OR N-Cov*[tiab] OR CV-19[tiab] OR CV19*[tiab] OR HCov*[tiab] OR Corono Virus[tiab] OR Virus Corona[tiab] OR Coronovir*[tiab] ORCoronavir*[tiab] OR (Pneumonia[tiab] AND Wuhan[tiab] AND 2019[tiab]) OR SARSCoV2[tiab] OR SARS-CoV2[tiab] OR SARS-CoV-2[tiab] OR 2019-nCoV[tiab] OR COVID19*[tiab] OR COVID-19[tiab] OR Corona Virus[tiab] COVID-19[Mesh] OR SARS-CoV-2[Mesh] Or Coronavirus[Mesh]) AND (Youth*[tiab] Teens[tiab] OR Teenage*[tiab] OR Adolescen*[tiab] OR Adolescent[Mesh] OR Preschool[tiab] ORBabies[tiab] ORToddler*[tiab] ORChild*[tiab] OR Paediatric*[tiab] OR Pediatric*[tiab] ORNewborn*[tiab] OR Neonat*[tiab] ORStill Birth*[tiab] OR Stillbirth*[tiab] OR Stillbirth[Mesh] OR Infant*[tiab] OR Infant[Mesh]) AND (Argentin*[ad] OR Argentina[pl] OR Argentin*[tiab] OR Argentina[Mesh])

EMBase (Ovid) 5/12/2022

Embase <1974 to 2022 December 02>

(exp Coronavirinae or exp Severe acute respiratory syndrome coronavirus 2 orexp coronavirus disease 2019or (Coron* adj1 Virus).ti,ab. orCOVID-19.ti,ab. COVID19*.ti,ab. Or 2019-nCoV.ti,ab. Or SARS-CoV-2.ti,ab. Or SARS-CoV2.ti,ab. or SARSCoV2.ti,ab. Or (Pneumonia and Wuhan and "2019").ti,ab. Or Coronavir*.ti,ab. or Coronovir*.ti,ab. Or HCov*.ti,ab. Or CV19*.ti,ab. Or CV-19.ti,ab. Or N-Cov*.ti,ab. Or Ncov*.ti,ab.) and (exp infant/ Infant*.ti,ab. exp stillbirth/ Stillbirth*.ti,ab. Or (Still adj1 Birth*).ti,ab. Or Neonat*.ti,ab. Or Newborn*.ti,ab. orPediatric*.ti,ab. Paediatric*.ti,ab. or Child*.ti,ab. Or Toddler*.ti,ab. Or Babies.ti,ab. Or Preschool.ti,ab. Or (Pre adj1 School).ti,ab. Or exp adolescent/ or Adolescen*.ti,ab. Or Teenage*.ti,ab. Or Teens.ti,ab. Or Youth*.ti,ab.) and exp (Argentina/ or Argentin*.mp.)

Web of Science 5/12/2022

(TS=Coronavirus OR TS=SARS-CoV-2 OR TS=COVID-19 OR TI=(Coron* NEAR/1 Virus) OR AB=(Coron* NEAR/1 Virus) OR TI=COVID-19 OR AB=Covid-19 OR TI=Covid19* OR AB=Covid19* OR TI=2019-nCoV OR AB=2019-nCoV OR TI=SARS-CoV-2 OR AB=SARS-CoV-2 OR TI=SARS-CoV2 OR AB=SARS-CoV2 OR TI=SARSCoV2 OR AB=SARSCoV2 OR TI=(Pneumonia AND Wuhan AND 2019) OR AB=(Pneumonia AND Wuhan AND 2019) OR TI=Coronavir* OR AB=Coronavir* OR TI=Coronovir* OR AB=Coronovir* OR TI=HCov* OR AB=HCov* OR TI=CV19* OR AB=CV19* OR TI=CV-19* OR AB=CV-19* OR TI=N-Cov* OR AB=N-Cov* OR TI=Ncov* OR AB=Ncov*) AND (TS=Infant OR TI=Infant* OR AB=Infant* OR TS=Stillbirth OR TI=Stillbirth* OR AB=Stillbirth* OR TI=Still-Birth* OR AB=Still-Birth* OR TI=Neonat* OR AB=Neonat* OR TI=Newborn* OR AB=Newborn* OR TI=Pediatric* OR AB=Pediatric* OR TI=Paediatric* OR AB=Paediatric* OR TI=Child* OR AB=Child* OR TI=Toddler* OR AB=Toddler* OR TI=Babies OR AB=Babies OR TI=Preschool OR AB=Preschool OR TI=Pre-School OR AB=Pre-School OR TS=Adolescent OR TI=Adolescen* OR AB=Adolesccen* OR TI=Teenage* OR AB=Teenage* OR TI=Teens OR AB=Teens

OR TI=Youth* OR AB=Youth*) AND (TS=Argentina OR TI=Argentin* OR AB=Argentin* OR AD=Argentin*)

LILACS 5/12/2022

(MH Coronavirus OR MH Coronavirus Infections OR Corona OR COVID-19$ OR COVID19$ OR 2019-nCoV OR SARS-CoV-2 OR SARS-CoV2 OR SARSCoV2 OR Coronavir$ OR Coronovir$ OR HCov$ OR CV19$ OR CV-19$ OR N-Cov$ OR NCov$) AND (MH Argentina OR Argentin$) AND (MH Infant OR Infant$ OR MH=Stillbirth OR Stillbirth$ OR Neonat$ OR Nacido$ OR Newborn$ OR Pediatric$ OR Paediatric$ OR Child$ OR Toddler$ OR Niños OR Meninos OR Crianca$ OR Babies OR Bebés OR Preschool OR Preescola$ OR MH Adolescent OR Adolescen$ OR Teenage$ OR Teens OR Youth$) [Words]

CINAHL (EBSCO) 5/12/2022

(TI NCov* OR AB NCov* or TI N-Cov* OR AB N-Cov*or TI CV-19* OR AB CV-19*or TI CV19* OR AB CV19*or TI HCov* OR AB HCov* or TI Coronavir* OR AB Coronavir* or TI (Pneumonia AND Wuhan AND 2019) OR AB (Pneumonia AND Wuhan AND 2019) or TI SARSCoV2 OR AB SARSCoV2 or TI SARS-CoV2 OR AB or SARS-CoV2 or TI SARS-CoV-2 OR AB SARS-CoV-2 or TI 2019-nCoV OR AB 2019-nCoV or TI COVID19* OR AB COVID19*or TI COVID-19* OR AB COVID-19* or TI (Coron* N1 Virus) OR AB (Coron* N1 Virus) or (MH "COVID-19+") or (MH "SARS-CoV-2") or (MH "Coronavirus+"))AND (TI Youth* OR AB Youth* OR TI Teens OR AB Teens OR TI Teenage* OR AB Teenage* TI Adolescen* OR AB Adolescen* OR (MH "Adolescence+") OR TI Pre-school OR AB Pre-school OR TI Preschool OR AB Preschool OR TI Babies OR AB Babies OR TI Toddler* OR AB Toddler* OR TI Child* OR AB Child* OR TI Paediatric* OR AB Paediatric* OR TI Pediatric* OR AB Pediatric* OR TI Newborn* OR AB Newborn* OR TI Neonat* OR AB Neonat* OR TI Still-birth* OR AB Still-birth* OR TI Stillbirth* OR AB Stillbirth* OR (MH "Perinatal Death") OR TI Infant* OR AB Infant* OR MH "Infant+") AND(TI Argentin* OR AB Argentin*)

EconLIT (Ovid) 5/12/2022

Econlit <1886 to November 24, 2022>

((Coron* adj1 Virus).ti,ab. OR COVID-19.ti,ab. OR COVID19*.ti,ab. OR 2019-nCoV.ti,ab. OR SARS-CoV-2.ti,ab. OR SARS-CoV2.ti,ab. OR SARSCoV2.ti,ab. OR (Pneumonia and Wuhan and "2019").ti,ab. OR Coronavir*.ti,ab.OR Coronovir*.ti,ab. OR HCov*.ti,ab. OR CV19*.ti,ab. OR CV-19.ti,ab OR N-Cov*.ti,ab. OR Ncov*.ti,ab.) AND (Infant*.ti,ab. OR Stillbirth*.ti,ab. OR (Still adj1 Birth*).ti,ab. OR Neonat*.ti,ab. OR Newborn*.ti,ab. OR Pediatric*.ti,ab. OR Paediatric*.ti,ab. OR Child*.ti,ab. OR Toddler*.ti,ab. OR Babies.ti,ab. OR Preschool.ti,ab. OR (Pre adj1 School).ti,ab. OR Adolescen*.ti,ab. OR Teenage*.ti,ab. OR Teens.ti,ab.) AND Argentin*.mp.

PsycINFO (Ovid) 6/12/2022

(exp Coronavirus/ or exp COVID-19/ or (Coron* adj1 Virus).ti,ab. Or COVID-19.ti,ab. Or COVID19*.ti,ab. Or 2019-nCoV.ti,ab. Or SARS-CoV-2.ti,ab. Or SARS-CoV2.ti,ab. Or SARSCoV2.ti,ab. Or (Pneumonia and Wuhan and "2019").ti,ab. Or Coronavir*.ti,ab. Or Coronovir*.ti,ab. Or HCov*.ti,ab. Or CV19*.ti,ab. Or CV-19*.ti,ab. Or N-Cov*.ti,ab. Or Ncov*.ti,ab.) AND (Infant*.ti,ab. Or Stillbirth*.ti,ab. Or Still Birth*.ti,ab. Or Neonat*.ti,ab. Or Newborn*.ti,ab. Or Pediatric*.ti,ab. Or Paediatric*.ti,ab. Or Child*.ti,ab. Or Toddler*.ti,ab. Or Babies.ti,ab. Or Preschool.ti,ab. Or Pre-School.ti,ab. Or Adolescen*.ti,ab. Or Teenage*.ti,ab. Or Teens.ti,ab. Or Youth*.ti,ab.) AND Argentin*.mp.

SciELO 6/12/2022

(COVID-19$ OR COVID19$ OR 2019-nCoV OR SARS-CoV-2 OR SARS-CoV2 OR SARSCoV2 OR Coronavir$ OR Coronovir$ OR HCov$ OR CV19$ OR CV-19$ OR N-Cov$ OR NCov$) AND Argentin$ AND (Infant$ OR Stillbirth$ OR Neonat$ OR Nacido$ OR Newborn$ OR Pediatric$ OR Paediatric$ OR Child$ OR Toddler$ OR Niños OR Meninos OR Crianca$ OR Babies OR Bebés OR Preschool OR Preescola$ OR Adolescen$ OR Teenage$ OR Teens OR Youth$)

Risk of bias

Table 1. Risk of bias for Case Series included in the systematic review

ID: Last Name Year of report	Study Type	1	2	3	4	5	6	7	8	9	Overall Rating
Aprea 2021	Case Series	Y	Y	Y	Y	NA	Y	NA	Y	Y	FAIR
Capra 2021	Case Series	Y	Y	Y	Y	NA	Y	NA	Y	Y	FAIR
Ferraro 2021	Case Series	Y	Y	Y	Y	NA	CD	NA	Y	Y	FAIR
Medina 2021	Case Series	N	N	Y	Y	NA	CD	NA	N	N	POOR
Raiden 2021	Case Series	Y	Y	Y	Y	NA	Y	NA	Y	Y	FAIR
Rubiños 2022	Case Series	Y	Y	Y	Y	NA	Y	Y	Y	Y	GOOD
Vainstein 2022	Case Series	Y	Y	Y	Y	NA	CD	Y	Y	Y	FAIR

Table 2. Risk of Bias for Cohort and Cross-Sectional Studies

Last Name Year of report	Study Type	1	2	3	4	5	6	7	8	9	10	11	12	13	14	Overall Rating
Brizuela 2022	Cohort	Y	Y	NR	Y	NR	NA	Y	Y	Y	CD	Y	N	Y	CD	FAIR
Gentile 2022	Cohort	Y	Y	Y	Y	NR	NR	Y	N	Y	N	Y	N	N	NR	GOOD
Gentile 2022 b.	Cohort	Y	Y	Y	Y	NR	NR	Y	N	Y	N	Y	N	N	NR	GOOD
Golemba 2022	Cross Sectional	Y	Y	N	Y	NR	NA	Y	Y	Y	N	Y	N	NA	NR	FAIR
Gómez 2021	Cohort	Y	Y	Y	Y	N	NA	NA	NA	Y	NR	Y	N	Y	N	POOR
Rosanova 2021	Cohort	Y	Y	Y	Y	N	NA	Y	N	Y	NR	Y	N	NR	Y	FAIR
Gentile 2023	Cohort	Y	Y	Y	Y	NR	NR	Y	N	Y	N	Y	N	N	NR	GOOD

Details on estimation of direct medical cost of COVID and MISC

In this section, we presented details on the estimation of direct medical costs of COVID and MISC used in the study. Table 3 details the doses per kg day of each drug included in the costing for each level of severity of COVID. These doses were calculated as recommended for a 55-kg adolescent.

Table 3. Drugs cost and dosage

	**Ex-Factory Price/mg, USD**	**Mild**	**Moderate**	**Severe**	**Critical**
Ibuprofen	$0.10	10 mg/kg/8hr/48hrs	10 mg/kg/8hr/48hrs	NA	NA
Ibuprofen IV	$0.10	NA	NA	NA	10 mg/kg/8hr/48hrs
Ampicillin	$0.80	NA	NA	150mg/kg/6hr/7days	150mg/kg/6h/7days
Ceftriaxone	$2.46	NA	NA	100 mg/kg/24hrs	100 mg/kg/24hrs
Vancomycin	$3.20	NA	NA	60mg/kg/6hr/7days	60mg/kg/6hr/7days
Dexamethasone	$18.46	NA	NA	0,15mg/kg/24hs/7days	0,15mg/kg/24hr/7days

To determine the level of severity of COVID-19 for the costing, we referred to the WHO Clinical Management Guide for COVID-19 [1], published in January 2021. This guide classified the severity of the disease (presented in the Table 6.3) as follows:

- Mild COVID-19: cases that require symptomatic treatment and do not present viral pneumonia or hypoxia.
- Moderate COVID-19: cases that require treatment for non-severe pneumonia.
- Severe COVID-19: patients with severe pneumonia.
- Critical COVID-19: patients with Acute Respiratory Distress Syndrome (ARDS).

Based on these definitions, we identified the healthcare resources that needed funding. The utilization rates of healthcare resources by the severity level of COVID are detailed in Table 4.

Table 4. Expected healthcare resource utilization (HCRU) for the treatment of COVID-19 case by level of severity

Resource	Mild*	Moderate*	Severe*°	Critical*°
Urgent consultation	0.6	0.6	0.4	0.6
Clinical medical consultation	0.4	0.4	0.6	0.4
Follow-up consultations	5	5	0	0
COVID-19 diagnosis	1	1	1	1
Admission in general ward	0	4.8	4	4
ICU admission without ventilatory support	0	0	3	3
ICU admission with ventilatory support	0	0	0	3
Electrocardiogram	0	0	0.8	0.8
Viral Antigens - extended PCR	0	0.5	0.5	0.5
Blood count	0	1	1	1
Hepatogram	0	1	1	1
ABG	0	1	1	1
Ionogram	0	1	1	1
Quantitative C Reactive Protein	0	0.5	0.5	0.5
Chest x-ray	0	2	2	2
Blood culture	0	1	1	1

*This severity level also includes medication for symptom management.
°This level of severity also includes antibiotics for the management of respiratory infection.

Supplementary Table 5 presents the unit costs of healthcare resources by healthcare sector in Argentina, expressed in USD 2023.

Table 5. Unit costs of healthcare resources used for the treatment of COVID-19 by healthcare sector in Argentina, expressed in USD 2023

Healthcare Resource	Public sector	Social security	Private sector
Urgent consultation	$3.86	$6.57	$10.46
Clinical medical consultation	$4.82	$8.19	$11.38
Follow-up consultations	$4.82	$8.19	$11.38
COVID-19 diagnosis	$16.24	$29.88	$31.68
Admission in general ward	$89.11	$220.99	$243.21
ICU admission without ventilatory support	$150.15	$372.36	$420.30
ICU admission with ventilatory support	$164.78	$408.65	$452.09
Electrocardiogram	$7.49	$8.47	$10.42
Viral Antigens - extended PCR	$112.06	$206.20	$218.57
Blood count	$1.46	$2.69	$2.85
Hepatogram	$2.92	$5.37	$5.69
ABG	$7.31	$13.45	$14.25
Ionogram	$1.71	$3.14	$3.33
Quantitative C Reactive Protein	$2.44	$4.48	$4.75
Chest x-ray	$9.42	$10.64	$12.07
Blood culture	$4.87	$8.96	$9.50

Table 6. Healthcare resource utilization (HCRU) and unit costs (USD 2023) per healthcare sector used for the treatment of MIS-C

Resource	HCRU	Public sector, $	Social security, $	Private sector, $
Admission in general ward	6	$89.11	$220.99	$243.21

ICU admission without ventilatory support	2.09	$150.15	$372.36	$420.30
ICU admission with ventilatory support	0.8987	$164.78	$408.65	$452.09
Rheumatology consultation	3	$6.21	$10.55	$12.46
Complete urine	1	$1.46	$2.69	$2.85
Blood culture	2	$4.87	$8.96	$9.50
CBC with platelet count	3	$1.46	$2.69	$2.85
C Reactive Protein	2	$2.44	$4.48	$4.75
TP/KPTT	1	$0.81	$1.49	$1.58
GOT/GPT	1	$0.73	$1.34	$1.43
LDH	1	$1.22	$2.24	$2.38
CPK	1	$4.87	$8.97	$9.50
Albuminemia	1	$0.84	$1.55	$1.64
Creatinine	1	$0.97	$1.79	$1.90
Ferritin	0.5	$7.31	$13.45	$14.25
BNP/NT-proBNP	0.2	$46.29	$85.17	$90.28
DimeroD	0.1	$17.05	$31.38	$33.26
Troponin	0.1	$8.28	$15.24	$16.16
Procalcitonin	1	$17.05	$31.38	$33.26
Fibrinogen	0.5	$8.28	$15.24	$16.16
Echocardiogram	2	$25.09	$28.35	$25.55

Detail of the research on the disease and economic burden of other immunopreventable diseases pre-vaccination era

The burden of immunopreventable diseases in Argentina prior to their incorporation into the vaccination schedule was substantial, necessitating attention and preventive measures. The following considered diseases—pneumococcal disease, meningococcal disease, influenza, rotavirus diarrhea, and chickenpox—caused a considerable impact on the population. Pneumococcal disease, caused by Streptococcus pneumoniae, emerged as a leading cause of bacteremia and bacterial meningitis among pediatric patients in Argentina. The disease exhibited a high mortality rate of 10% in cases of meningitis. Notably, children under five years old experienced a considerable burden, with an estimated incidence of 1,256 cases per

100,000 inhabitants for consolidating pneumonia. Hospitalization rates for pneumonia reached approximately 65%. The overall mortality rate for pneumonia in this age group was reported as 1.1%. Furthermore, pneumococcal bacteremia and sepsis affected children under two years old, with an incidence rate of 98.6 cases per 100,000 inhabitants and a lethality rate of 1.5%. Pneumococcal meningitis had an incidence of 3.49 cases per 100,000 children under five, with an associated mortality rate of approximately 14%.

Meningococcal disease posed a significant burden in Argentina, primarily affecting children under five years of age. Infants under 12 months old were particularly vulnerable, experiencing an incidence rate of 13.2 cases per 100,000 inhabitants. Notably, infants under nine months accounted for 64% of the affected population. The disease resulted in an annual average of 20 to 30 deaths, with a case fatality rate of 10%. Serogroups B and W were identified as the predominant strains circulating in Argentina, constituting 91% of Neisseria meningitidis isolates.

The inclusion of influenza vaccination in Argentina's National Immunization Schedule targeted specific high-risk groups due to the substantial impact of the disease. A retrospective evaluation conducted between 2002 and 2009 demonstrated excess mortality and hospitalizations associated with influenza. During this period, 2,411 deaths were attributed to pneumonia and influenza, while 8,527 deaths were coded as respiratory and circulatory diseases related to influenza. Hospitalization rates were estimated at 7,868 per year with pneumonia and influenza codes and 22,994 per year with respiratory and circulatory disease codes. The annual incidence rate of influenza-like illness was estimated at 36,080 cases per 100,000 individuals.

Rotavirus diarrhea emerged as a significant public health concern in Argentina. Annually, the country reported approximately 1,100,000 to 1,250,000 cases of acute diarrhea, with children under five accounting for 45-50% of the burden. The incidence rates in this age group ranged from 1,450 to 1,650 cases per 10,000 individuals. The severity of rotavirus diarrhea depended on socioeconomic and cultural factors as well as proximity to primary healthcare centers. The NOA and Cuyo regions exhibited the highest rates of acute diarrhea, with the NOA region reporting an annual rate of 3,500 cases per 10,000 children under five. Hospitalizations due to infectious diarrhea among children under five were estimated at 22,000 to 27,000 per year, with infants under one year old accounting for 30 to 35% of cases. Acute diarrhea represented approximately 10% of hospitalizations during early childhood and contributed to 60 to 130 deaths annually, accounting for 1.2% of deaths among children under five.

Mortality

The following ICD-10 codes and definitions were used to filter the local databases for deaths related to the immunopreventable diseases of interest:

Pneumococcal disease: “Sepsis debida a Streptococcus pneumoniae”,“Meningitis neumocócica”,“Neumonía debida a Streptococcus pneumoniae”,“Neumonía bacteriana, no especificada” , “Neumonía lobar, no especificada”: "J159" "A403" "G001" "J13X" "J181"

Meningococcal disease: “Meningitis meningocócica” , “Meningococemia, no especificada”: "A390" "A394"

Figure 1: Cumulative deaths by year of the other immunopreventable diseases of interest (Pneumococcal disease, Meningococcal disease and Influenza)

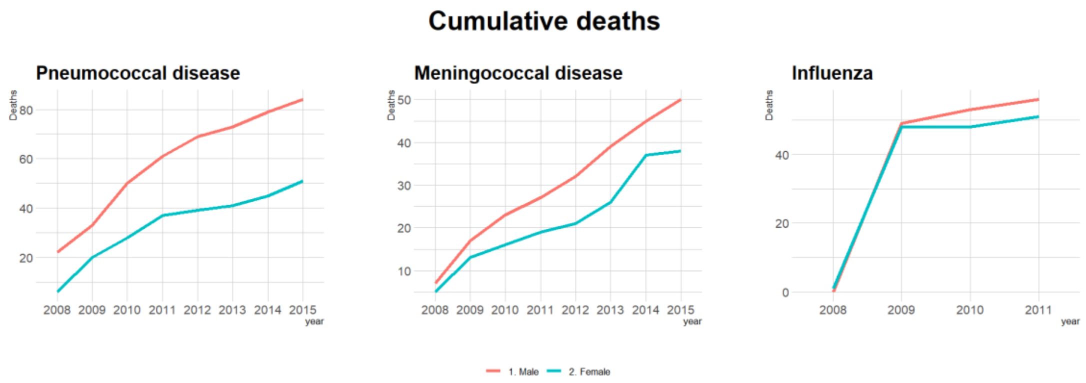

For cost data extraction, a literature search was conducted using the PubMed and Lilacs databases to estimate the costs associated with pneumococcal disease, influenza, meningococcal disease, rotavirus, and chickenpox. Studies of economic burden or direct medical costs were identified for each vaccine-preventable disease in Argentina. Four studies for pneumococcus, four for rotavirus, and two for influenza were included. In the particular case of pneumococcal disease, since not all studies had the same definition for pneumococcal disease, we decided to take all invasive diseases defined as consolidated pneumonia, focal pneumonia, meningitis, and bacteremia/sepsis as pneumococcal disease for our extraction of costs. From each study, the number of cases reported for each type of pneumococcal disease was taken and we performed a weighted average, so that we were able to obtain a value per case of pneumococcal disease. No economic studies of meningococcus were found in the country. The costs were updated for inflation to April 2023. Official data from the Banco de la República of Argentina was used to adjust for inflation [2]. Costs are reported in US dollars for the year 2023[3]. The search strategies used, the databases and the results obtained from them are detailed below.

PubMed
Pneumococcal Disease

(Pneumococcal Infections[Mesh] OR Pneumococc*[tiab] OR Streptococc*[tiab]) AND (Value of Life[Mesh] OR Economics, Dental[Mesh] OR Economics, Hospital[Mesh] OR Economics, Medical[Mesh] OR Economics, Pharmaceutical[Mesh] OR Economics, Nursing[Mesh] OR “Fees and Charges”[Mesh] OR Budgets[Mesh] OR Models, Economic[Mesh] OR “Costs and Cost Analysis”[Mesh] OR Cost[tiab] OR Costs[tiab] OR Costed[tiab] OR Costly[tiab] OR Economic*[ti] OR Pharmacoeconomic*[tiab] OR Price*[tiab] OR Pricing[tiab] OR Contingent Valuat*[tiab] OR “Willingness to Pay”[tiab] OR Conjoint Analysis[tiab] OR DALY*[tiab] OR QALY*[tiab] OR Burden[tiab] OR Quality-Adjusted[tiab] OR Expenditure*[tiab] OR Out-of-Pocket[tiab] OR Health Resources[Mesh]) AND (Argentina[Mesh] OR Argentin*[tiab])

Results: 26 titles
Excluded by title and abstract: 10 studies
For full review: 6 studies
Excluded studies: 2 studies
Included studies: 4 studies

Meningococcal Disease
(Meningococcal Infections[Mesh] OR Meningococc*[tiab]) AND (Value of Life[Mesh] OR Economics, Dental[Mesh] OR Economics, Hospital[Mesh] OR Economics, Medical[Mesh] OR Economics, Pharmaceutical[Mesh] OR Economics, Nursing[Mesh] OR “Fees and Charges”[Mesh] OR Budgets[Mesh] OR Models, Economic[Mesh] OR “Costs and Cost Analysis”[Mesh] OR Cost[tiab] OR Costs[tiab] OR Costed[tiab] OR Costly[tiab] OR Economic*[ti] OR Pharmacoeconomic*[tiab] OR Price*[tiab] OR Pricing[tiab] OR Contingent Valuat*[tiab] OR “Willingness to Pay”[tiab] OR Conjoint Analysis[tiab] OR DALY*[tiab] OR QALY*[tiab] OR Burden[tiab] OR Quality-Adjusted[tiab] OR Expenditure*[tiab] OR Out-of-Pocket[tiab] OR Health Resources[Mesh]) AND (Argentina[Mesh] OR Argentin*[tiab])

Results: 4 titles
Excluded by title and abstract: 4 studies
For full review: 0 studies
Excluded studies: 0 stidies
Included studies: 0 studies

Influenza
(Influenza, Human[Mesh] OR Influenza[tiab] OR Flu[tiab] OR Grippe[tiab]) AND (Value of Life[Mesh] OR Economics, Dental[Mesh] OR Economics, Hospital[Mesh] OR Economics, Medical[Mesh] OR Economics, Pharmaceutical[Mesh] OR Economics, Nursing[Mesh] OR “Fees and Charges”[Mesh] OR Budgets[Mesh] OR Models, Economic[Mesh] OR “Costs and Cost Analysis”[Mesh] OR Cost[tiab] OR Costs[tiab] OR Costed[tiab] OR Costly[tiab] OR Economic*[ti] OR Pharmacoeconomic*[tiab] OR Price*[tiab] OR Pricing[tiab] OR Contingent Valuat*[tiab] OR “Willingness to Pay”[tiab] OR Conjoint Analysis[tiab] OR DALY*[tiab] OR QALY*[tiab] OR Burden[tiab] OR Quality-Adjusted[tiab] OR Expenditure*[tiab] OR Out-of-Pocket[tiab] OR Health Resources[Mesh]) AND (Argentina[Mesh] OR Argentin*[tiab])

Results: 28 titles
Excluded by title and abstract: 22 studies
For full review: 6 studies
Excluded studies: 2 studies
Included studies: 4 studies

Rotavirus

(Rotavirus Infections[Mesh] OR Rotavirus[tiab]) AND (Value of Life[Mesh] OR Economics, Dental[Mesh] OR Economics, Hospital[Mesh] OR Economics, Medical[Mesh] OR Economics, Pharmaceutical[Mesh] OR Economics, Nursing[Mesh] OR “Fees and Charges”[Mesh] OR Budgets[Mesh] OR Models, Economic[Mesh] OR “Costs and Cost Analysis”[Mesh] OR Cost[tiab] OR Costs[tiab] OR Costed[tiab] OR Costly[tiab] OR Economic*[ti] OR Pharmacoeconomic*[tiab] OR Price*[tiab] OR Pricing[tiab] OR Contingent Valuat*[tiab] OR “Willingness to Pay”[tiab] OR Conjoint Analysis[tiab] OR DALY*[tiab] OR QALY*[tiab] OR Burden[tiab] OR Quality-Adjusted[tiab] OR Expenditure*[tiab] OR Out-of-Pocket[tiab] OR Health Resources[Mesh]) AND (Argentina[Mesh] OR Argentin*[tiab])

Results: 21 titles
Excluded by title and abstract: 15 studies
For full review: 6 studies
Excluded studies: 2 studies
Included studies: 4 studies

Chickenpox
(Chickenpox[Mesh] OR Chickenpox[tiab] OR Varicel*[tiab]) AND (Value of Life[Mesh] OR Economics, Dental[Mesh] OR Economics, Hospital[Mesh] OR Economics, Medical[Mesh] OR Economics, Pharmaceutical[Mesh] OR Economics, Nursing[Mesh] OR “Fees and Charges”[Mesh] OR Budgets[Mesh] OR Models, Economic[Mesh] OR “Costs and Cost Analysis”[Mesh] OR Cost[tiab] OR Costs[tiab] OR Costed[tiab] OR Costly[tiab] OR Economic*[ti] OR Pharmacoeconomic*[tiab] OR Price*[tiab] OR Pricing[tiab] OR Contingent Valuat*[tiab] OR “Willingness to Pay”[tiab] OR Conjoint Analysis[tiab] OR DALY*[tiab] OR QALY*[tiab] OR Burden[tiab] OR Quality-Adjusted[tiab] OR Expenditure*[tiab] OR Out-of-Pocket[tiab] OR Health Resources[Mesh]) AND (Argentina[Mesh] OR Argentin*[tiab])

Results: 8 titles
Excluded by title and abstract: 5 studies
For full review: 6 studies
Excluded studies: 3 studies
Included studies: 2 studies

Lilacs
(MH Pneumococcal Infections OR Pneumococ$ OR Neumococ$ OR Streptococ$ OR Estreptococ$ OR MH Influenza, Human OR Influenza OR Flu OR Grippe OR Gripe OR MH Meningococcal Infections OR Meningococ$ OR MH Chickenpox OR Chickenpox OR Catapora OR Varicel$ OR MH Rotavirus Infections OR Rotavirus) AND (MH Value of Life OR MH Economics, Dental OR MH Economics, Hospital OR MH Economics, Medical OR MH Economics, Pharmaceutical OR MH Economics, Nursing OR MH Budgets OR MH Models, Economic OR MH Costs and Cost Analysis OR Cost OR Costs OR Costed OR Costo OR Costos OR Custo OR Costly OR TI Economic$ OR Pharmacoeconomic$ OR Farmacoeconom$ OR Price$ OR Precio$ OR Preço$ OR Pricing OR Contingent$ OR ((Willingness OR Voluntad OR Vontade OR Disposición) AND (Pay OR Pago OR Pagar)) OR Conjoint OR DALY$ OR QALY$ OR Burden OR Carga OR Quality-Adjusted OR Expenditure$ OR Gasto$ OR Despesa$ OR Out-of-Pocket OR MH Health Resources) AND (MH Argentina OR Argentin$)

Results: 35 titles
Excluded by title and abstract: 28 studies
For full review: 7 studies
Excluded studies: 7 studies
Included studies: 0 studies

Forest plots showing proportion meta-analyses (N=14 studies) of demographics, clinical

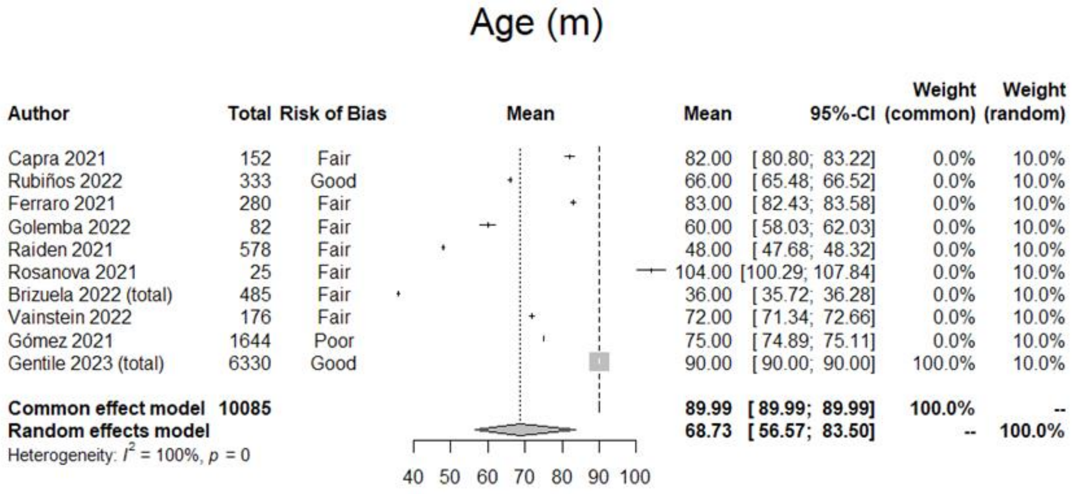

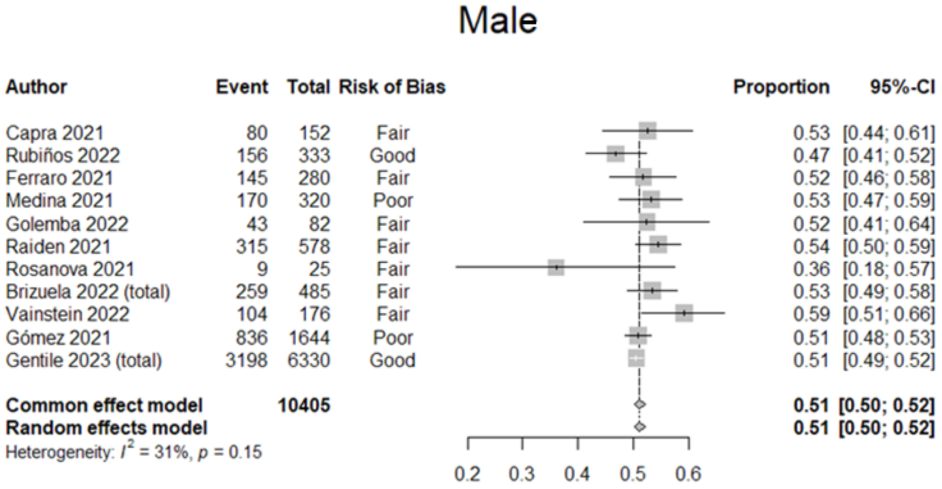

Underlying diseases

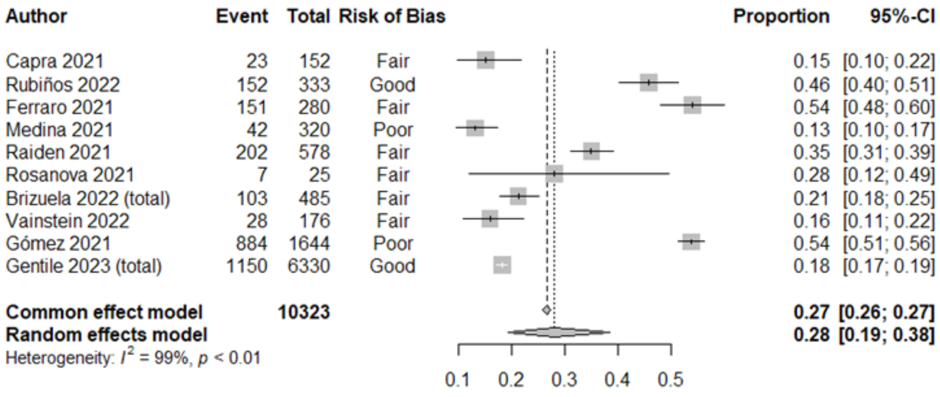

MIS-C/Kawasaki total

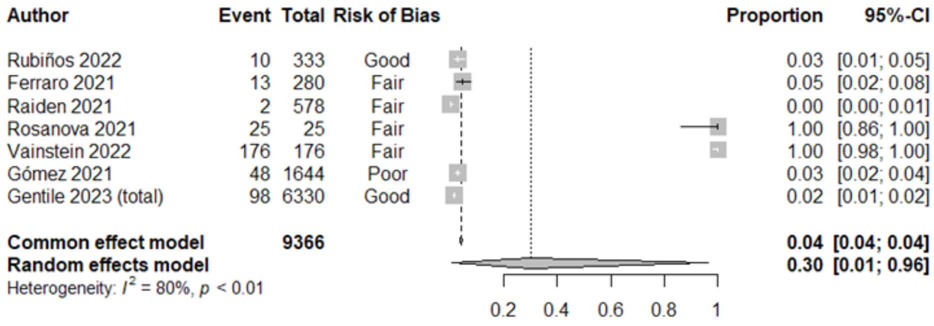

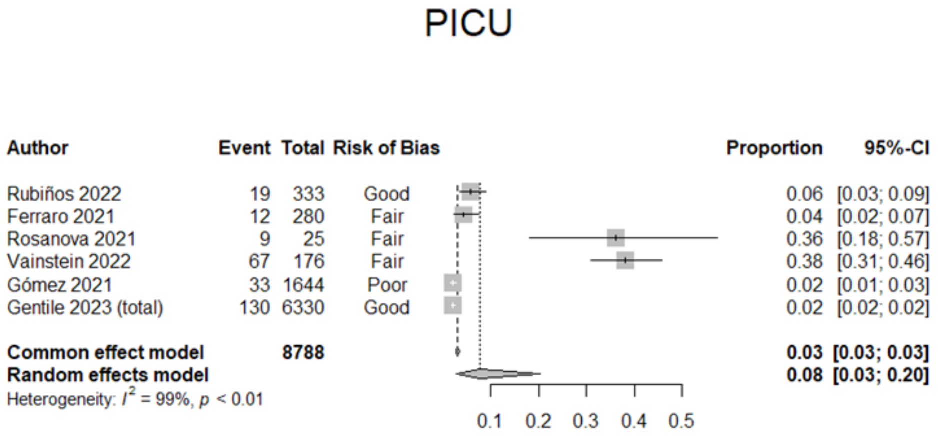

Table 8. Symptoms and comorbidities

Results of the pooled meta-analysis of the considered studies considering symptoms and comorbidities associated with COVID-19 in children and adolescents.

Variables	Studies (*) n/N	Cases/ Total n/N	Pooled proportion (95%CI) from meta-analyses
Gastrointestinal symptoms	11/14	2013/10444	15 (8-25)
Respiratory symptoms	11/14	4820/10444	31 (21-42)
Fever	11/14	6265/10444	60(45-73)
Neurological symptoms	8/14	1796/9366	10(6-18)
Rash	6/14	101/2999	5(1-16)
Comorbidity: Oncohematological	8/14	398/9743	5(2-10)
Comorbidity: Neurological	8/14	327/9190	4(3-7)
Comorbidity: Respiratory	9/14	759/9768	6 (4-8)
Comorbidity: Genetic	4/14	26/1169	2 (1-4)
Comorbidity: Others	7/14	537/7791	6 (3-11)

Total economic burden of COVID-19 case by healthcare sector

Table 9 presents the economic burden associated with COVID by healthcare sector and level of severity of COVID.

Table 9. Total economic burden of COVID-19 case by healthcare sector, expressed in USD 2023

	Public sector	Social Security	Private sector	National
**Mild COVID-19**	$7,138,591.50	$14,904,591.00	$6,622,024.90	$28,665,207.40
**Moderate COVID-19**	$2,287,551.70	$6,294,294.50	$2,420,653.70	$11,002,499.90
**Severe COVID-19**	$5,048,521.60	$12,106,125.70	$4,620,156.80	$21,774,804.20
**Critical COVID-19**	$4,176,736.40	$10,603,900.40	$4,054,335.70	$18,834,972.50
**All COVID-19**	$18,651,401.20	$43,908,911.60	$17,717,171.10	$80,277,484.00
**MIS-C**	$4,213,666.48	$5,921,177.54	$5,986,200.37	$5,282,726.99
**Total**	$41,516,468.88	$93,739,000.74	$41,420,542.57	$165,837,694.99

Results of other immunopreventable diseases pre-vaccination era

Table 10. Deaths of other immunopreventable diseases pre-vaccination era

	Influenza (2008-2010)	Meningococcal disease (2009-2015)	Pneumococcal Disease (2010)	Varicella (2008-2012)	Rotavirus (2008-2011)	COVID19 (2020-2021)
Average Deaths per year	35	13	389	19,4	97.25**	158

*Meningitis, sepsis and consolidated pneumonia

**Acute diarrhea

Table 11 presents the results of the costs of other inmunopreventable diseases pre- vaccination era collected from each study, updated to 2023 and expressed in USD 2023.

Table 11. Costs of other immunopreventable diseases pre-vaccination era, expressed in USD 2023

	**Cost per pneumococcus case**	
Augustovski, 2009	$2,028.16	
Giglio, 2010	$1,026.13	
Ureña, 2011	$1,382.08	
García-Martí, 2013	$1,898.76	
	**Cost per rotavirus case**	
García-Martí, 2015	$41.99	
Ureña, 2015	$36.48	
Giglio, 2017	$96.30	
García-Martí, 2019	$87.99	
	**Cost per hospitalized influenza case**	
Dayan, 2001	$344.40	
Marcone, 2015	$623.23	
	**Cost per outpatient chickenpox case**	**Cost per inpatient chickenpox case**
Bardach, 2012	$71.55	$2,085.31
Giglio, 2018	$47.26	$721.58

References

1. OMS. Manejo clínico de la COVID-19: orientaciones evolutivas, 25 de enero de 2021. In: OMS [Internet]. Available: https://iris.who.int/handle/10665/340629?&locale-attribute=pt

2. INDEC, Instituto Nacional de Estadistica y Censos de la REPUBLICA ARGENTINA. INDEC: Instituto Nacional de Estadística y Censos de la República Argentina. [cited 21 Jun 2023]. Available: https://www.indec.gob.ar/

3. Tipos de Cambio BCRA. In: Banco Centra de la República de Argentina [Internet]. [cited 21 Jun 2023]. Available: https://www.bcra.gob.ar/PublicacionesEstadisticas/Tipos_de_cambios.asp
